# TRIM3 attenuates apoptosis in Parkinson's disease via activating PI3K/AKT signal pathway

**DOI:** 10.18632/aging.202181

**Published:** 2020-11-30

**Authors:** Wenwen Dong, Bei Luo, Chang Qiu, Xu Jiang, Bo Shen, Li Zhang, Weiguo Liu, Wenbin Zhang

**Affiliations:** 1 Department of Functional Neurosurgery, The Affiliated Brain Hospital of Nanjing Medical University, Nanjing 210029, Jiangsu Province, China; 2 Neurology Department, The Affiliated Brain Hospital of Nanjing Medical University, Nanjing 210029, Jiangsu Province, China

**Keywords:** PD, TRIM3, apoptosis, ROS, PI3K/AKT

## Abstract

This article aims to study tripartite motif-containing protein 3 (TRIM3) effects on Parkinson's disease (PD). TRIM3 expression in venous blood of PD patients was detected by qRT-PCR. PD mouse model and PD SH-SY5Y cell model were constructed. PD cells were treated by LY294002 (a PI3K inhibitor). The apoptosis of PD mouse midbrain was detected. Glutathione (GSH) and superoxide dismutase (SOD) level in PD cells and mice midbrain was analyzed. Intracellular reactive oxygen species (ROS) and MMP were detected. The effect of TRIM3 on cell viability, apoptosis and PI3K/AKT signal pathway were analyzed. As a result, TRIM3 expression in venous blood of PD patients was decreased. TRIM3 up-regulation in PD mouse decreased midbrain tissues apoptosis. TRIM3 up-regulation increased GSH and SOD levels in PD mice midbrain tissues and PD cells. TRIM3 up-regulation in PD cells prominently reduced ROS and MMP. TRIM3 up-regulation increased PD cells viability and decreased apoptosis. TRIM3 up-regulation in PD cells elevated Bcl-2 protein expression and weakened Bax, Cleaved-caspase 3 and Cleaved-caspase 9 proteins expression. TRIM3 up-regulation increased p-PI3K/PI3K and p-AKT/AKT ratio. PI3K inhibitor treatment reversed the inhibitory effect of TRIM3 up-regulation on PD cells apoptosis. Thus, TRIM3 might attenuate apoptosis in PD via activating PI3K/AKT signal pathway.

## INTRODUCTION

Parkinson's disease (PD) is a common neurodegenerative disease accompanied by a major clinical manifestation of motor dysfunction, such as postural instability, static tremor, rigidity and cognition disorder [1, 2]. PD, which often occurs in people over 65 years of age, is caused by the progressive degradation of dopaminergic neurons in the pars compacta region of substantia nigra [3]. PD brings great inconvenience to patients' quality of life. To date, PD is incurable despite different preventative strategies, such as anti-inflammatory drugs, anti-oxidants and physical therapy which can alleviate PD-related symptoms to some extent [4]. The elucidation of PD pathogenesis is of great significance for the development of effective treatment strategies.

At present, the pathogenesis of PD has attracted widespread attention in molecular biology. The discovery of PD pathogenesis at the molecular level provides effective therapeutic targets for the treatment of PD. In addition to non-coding RNAs (including microRNAs and long-chain non-coding RNA) [5, 6], several important protein-coding genes have also been found to be involved in the occurrence and regulation of PD. For instance, human Ndfipl was considered to have neuroprotective effect, because Ndfipl could reduce apoptosis and increase cell viability of PD cell model in vitro [7]. Researchers also noticed that, SIRT1 was conductive to protection of salidroside against apoptosis and oxidative stress in PD cell models in vitro. The mechanism might be through partially inhibiting the mitogen-activated protein kinase pathway [8]. Liu et al. [9] reported that, the up-regulation of CDKN2D in PD rat model could suppress the apoptosis of substantia nigra dopaminergic neurons. With the development of molecular biotechnology and the progression of clinical treatment strategies, these findings will provide great possible and effective targets for the complete cure of PD.

We previously screened for biomarkers of PD using proteomics and bioinformatics approaches [10]. Quantitative proteomic analysis of human sera was performed through the use of tandem mass tag markers and liquid chromatography-mass spectrometry (LC-MS)-based techniques. We reported that tripartite motif-containing protein 3 (TRIM3) was one of the key proteins involved in the molecular mechanisms of PD [10]. TRIM3 belongs to the TRIM family, which is located on chromosome 11p15.5 and has been confirmed as a tumor suppressor [11]. However, the function of TRIM3 in PD has never been defined. A previous study reported that TRIM9 was a homologous protein of TRIM3 and was severely reduced in the mouse brain areas in PD and dementia with Lewy bodies [12]. Based on this, we speculated that TRIM3 might be involved in the regulation of PD. Thus, the expression and impact of TRIM3 in PD was investigated in this paper. Besides, the PI3K/AKT signal pathway was demonstrated to mediate the neuroprotection in PD [13]. Therefore, the association between TRIM3 and PI3K/AKT signal pathway in PD was further explored in the present study. This study will provide new insight and target for the treatment of PD.

## RESULTS

### TRIM expression was decreased in PD patients, PD mice model and cell model

**Figure 1:**
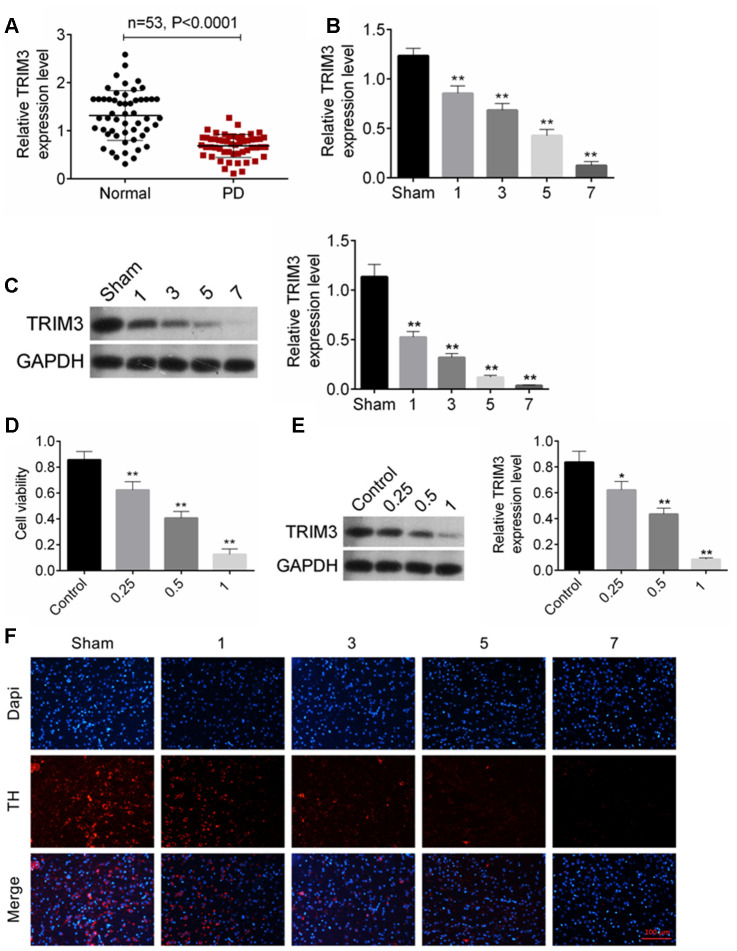
**TRIM expression was decreased in PD patients, PD mice and cells.** (**A**) TRIM3 mRNA expression in venous blood samples of 53 patients with PD was reduced. (**B** and **C**) MPTP-HCl injection prominently decreased TRIM3 mRNA and protein expression in the mice midbrain with a time-dependent manner. (**D**) MPP^+^ treatment dramatically reduced cells viability with a dose-dependent manner. (**E**) MPP^+^ treatment significantly decreased TRIM3 protein expression in SH-SY5Y cells with a dose-dependent manner. (**F**) MPP^+^ treatment obviously reduced the number of TH positive expression dopaminergic neurons in the mice midbrain with a time-dependent manner. * *P* < 0.05 and ** *P* < 0.01 relative to Sham group or Control group.

The expression of TRIM3 mRNA in venous blood samples from 53 patients with PD and 53 healthy subjects was detected by qRT-PCR. The results showed that, compared to 53 healthy subjects, the relative TRIM3 mRNA expression in venous blood samples of 53 patients with PD was remarkably decreased (*P* < 0.0001) (Figure 1A). PD mouse model was constructed by injection of MPTP-HCl. Relative to Sham group, MPTP-HCl injection prominently decreased TRIM3 mRNA and protein expression in the mice midbrain with a time-dependent manner (*P* < 0.01) (Figure 1B, 1C). In addition, relative to SH-SY5Y cells in Control group, MPP^+^ treatment dramatically reduced cells viability and TRIM3 protein expression with a dose-dependent manner (*P* < 0.01 or *P* < 0.05) (Figure 1D, 1E). The number of dopaminergic neurons was evaluated by detecting TH expression using immunofluorescence. As shown in Figure 1F, compared with Sham group, MPTP-HCl injection obviously reduced the number of TH positive expression (red immunofluorescence) dopaminergic neurons in a time-dependent manner.

### TRIM3 inhibited apoptosis of brain tissue cells in PD mice

The pole test score of PD mice was measured on the 1, 2, 3, 4 and 5 days. As shown in Table 1, in comparison with Sham group, mice of MPTP group and MPTP + oeNC group had obviously lower pole test score during 1-5 days (*P* < 0.01). However, relative to MPTP + oeNC group, the pole test score of mice in MPTP + oeTRIM3 group was much higher (*P* < 0.05). Thus, TRIM3 overexpression improved the symptom of bradykinesia in PD mice.

**Table 1:** Pole test score of mice.

**Groups**	**Days**				
	**1d**	**2d**	**3d**	**4d**	**5d**
Sham	7.13 ± 1.52	7.95 ± 1.37	8.13 ± 1.45	8.74 ± 1.65	9.13 ± 1.98
MPTP	3.47 ± 1.38**	3.93 ± 1.42**	4.12 ± 1.28**	4.57 ± 1.26**	4.86 ± 1.32**
MPTP + oeNC	3.51 ± 1.34**	4.13 ± 1.27**	4.36 ± 1.41**	4.48 ± 1.46**	5.12 ± 1.62**
MPTP + oeTRIM3	4.69 ± 1.29^#^	4.81 ± 1.38^#^	5.23 ± 1.43^#^	6.12 ± 1.56^#^	6.79 ± 1.71^#^

** *P* < 0.01 relative to Sham group. # *P* < 0.05 relative to MPTP + oeNC group.

**Figure 2:**
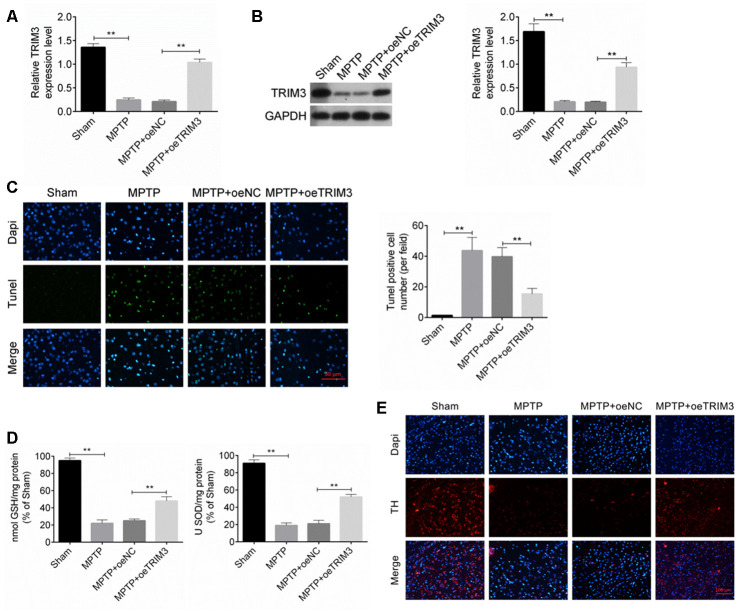
**TRIM3 inhibited apoptosis of brain tissue cells in PD mice.** (**A** and **B**) TRIM3 mRNA and protein expression in the midbrain of mice in MPTP + oeTRIM3 group was significantly elevated when compared with MPTP + oeNC group. (**C**) Markedly lower Tunel positive cell number was found in the midbrain of mice in MPTP + oeTRIM3 group relative to MPTP + oeNC group. (**D**) Prominently higher GSH and SOD levels were observed in the midbrain of mice in MPTP + oeTRIM3 group when compared with MPTP + oeNC group. (**E**) Compared with MPTP + oeNC group, more TH positive expression dopaminergic neurons were observed in MPTP + oeTRIM3 group. ** *P* < 0.01.

TRIM3 mRNA and protein expression in mice midbrain was detected by qRT-PCR and Western blot. As shown in Figure 2A, 2B, relative to Sham group, TRIM3 mRNA and protein expression in the MPTP group was remarkably decreased (*P* < 0.01). However, when compared with the TRIM3 mRNA and protein expression in mice of MPTP + oeNC group, it was significantly elevated in the midbrain of the MPTP + oeTRIM3 group (*P* < 0.01).

Tunel assay was performed to research the apoptosis of midbrain cells. Mice of MPTP group exhibited much higher Tunel positive cell number than that of Sham group (*P* < 0.01). Markedly lower Tunel positive cell number was found in MPTP + oeTRIM3 group relative to MPTP + oeNC group (*P* < 0.01) (Figure 2C). Detection of GSH and SOD levels in the midbrain showed that, compared with Sham group, significantly lower GSH and SOD levels was occurred in mice of MPTP group (*P* < 0.01). On the opposite, prominently higher GSH and SOD levels was observed in mice of MPTP + oeTRIM3 group when compared with MPTP + oeNC group (*P* < 0.01) (Figure 2D). Immunofluorescence result exhibited that relative to Sham group, obviously less TH positive expression (red immunofluorescence) dopaminergic neurons were found in MPTP group. Conversely, compared with MPTP + oeNC group, more TH positive expression dopaminergic neurons were observed in MPTP + oeTRIM3 group (Figure 2E).

### TRIM3 relieved oxidative stress in PD cell model

**Figure 3:**
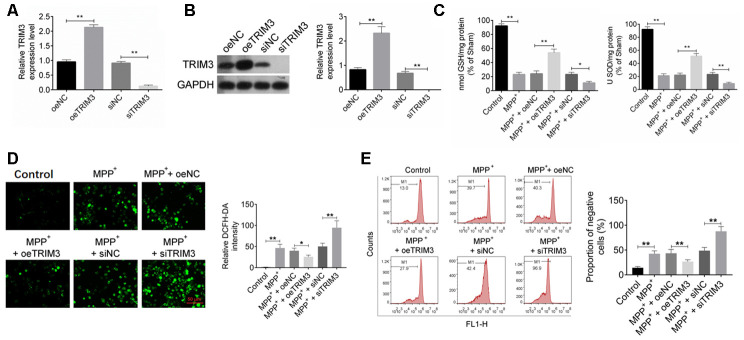
**TRIM3 relieved oxidative stress in PD cell model.** (**A** and **B**) TRIM3 mRNA and protein expression in SH-SY5Y cells was successfully regulated by transfection. (**C**) TRIM3 significantly elevated the level of GSH and SOD in SH-SY5Y cells induced by MPP^+^ solution. (**D**) TRIM3 markedly reduced the level of ROS in SH-SY5Y cells induced by MPP^+^ solution. (**E**) TRIM3 prominently decreased the MMP in SH-SY5Y cells induced by MPP^+^ solution. * *P* < 0.05 and ** *P* < 0.01.

As shown in Figure 3A, 3B, SH-SY5Y cells of oeTRIM3 group exhibited much higher TRIM3 mRNA and protein expression than that of oeNC group (*P* < 0.01). Conversely, SH-SY5Y cells of siTRIM3 group showed markedly lower TRIM3 mRNA and protein expression that that of siNC group (*P* < 0.01). Thus, SH-SY5Y cells were successfully transfected.

By treatment with 1 mM MPP^+^ solution, GSH and SOD level in SH-SY5Y cells of MPP^+^ group was significantly reduced when compared with Control group (*P* < 0.01). Furthermore, GSH and SOD level in SH-SY5Y cells of MPP^+^ + oeTRIM3 group was prominently higher than that in MPP^+^ + oeNC group (*P* < 0.01). However, relative to MPP^+^ + siNC group, the GSH and SOD level in SH-SY5Y cells of MPP^+^ + siTRIM3 group was dramatically reduced (*P* < 0.01) (Figure 3C). The intracellular ROS in SH-SY5Y cells of each group were measured respectively. As shown in Figure 3D, compared with Control group, cells of MPP^+^ group had obviously higher relative DCFH-DA intensity (*P* < 0.01). However, relative to MPP^+^ + oeNC group, much lower relative DCFH-DA intensity was found in cells of MPP^+^ + oeTRIM3 group (*P* < 0.05). SH-SY5Y cells of MPP^+^ + siTRIM3 group showed markedly higher relative DCFH-DA intensity than that of MPP^+^ + siNC group (*P* < 0.01). The MMP of SH-SY5Y cells were further detected. The results were presented in Figure 3E. Relative to Control group, SH-SY5Y cells of MPP^+^ group showed much higher negative cells proportion (*P* < 0.01). Conversely, SH-SY5Y cells of MPP^+^ + oeTRIM3 group exhibited obviously lower negative cells proportion than that of MPP^+^ + oeNC group (*P* < 0.01). In comparison with MPP^+^ + siNC group, markedly higher negative cells proportion was observed in SH-SY5Y cells of MPP^+^ + siTRIM3 group (*P* < 0.01).

### TRIM3 attenuated apoptosis in PD cell model via activating PI3K/AKT signal pathway

**Figure 4:**
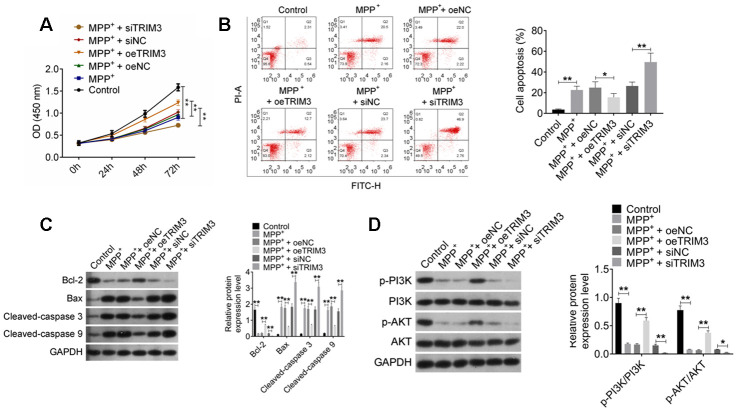
**TRIM3 attenuated apoptosis in PD cell model via activating PI3K/AKT signal pathway.** (**A**) TRIM3 promoted proliferation of SH-SY5Y cells induced by MPP^+^ solution. (**B**) TRIM3 attenuated apoptosis of SH-SY5Y cells induced by MPP^+^ solution. (**C**) TRIM3 elevated Bcl-2 protein expression and reduced Bax, Cleaved-caspase 3 and Cleaved-caspase 9 proteins expression in SH-SY5Y cells induced by MPP^+^ solution. (**D**) TRIM3 increased the ratio of p-PI3K/PI3K and p-AKT/AKT in SH-SY5Y cells induced by MPP^+^ solution. * *P* < 0.05 and ** *P* < 0.01.

As shown in Figure 4A, 4B, SH-SY5Y cells of MPP^+^ group had much lower OD value and higher apoptosis percentage than that of Control group (*P* < 0.01). Meanwhile, compared with MPP^+^ + oeNC group, obviously higher OD value and lower apoptosis percentage was observed in SH-SY5Y cells of MPP^+^ + oeTRIM3 group (*P* < 0.01 or *P* < 0.05). When relative to MPP^+^ + siNC group, SH-SY5Y cells of MPP^+^ + siTRIM3 group had remarkably lower OD value and higher apoptosis percentage (*P* < 0.01).

This research analyzed the expression of important apoptosis-related proteins in SH-SY5Y cells. As a result, SH-SY5Y cells of MPP^+^ group exhibited significantly lower Bcl-2 protein expression and higher Bax, Cleaved-caspase 3 and Cleaved-caspase 9 proteins expression than that of Control group (*P* < 0.01). SH-SY5Y cells of MPP^+^ + oeTRIM3 group had much higher Bcl-2 protein expression and lower Bax, Cleaved-caspase 3 and Cleaved-caspase 9 proteins expression than that of MPP^+^ + oeNC group (*P* < 0.01). However, prominently lower Bcl-2 protein expression and higher Bax, Cleaved-caspase 3 and Cleaved-caspase 9 proteins expression was occurred in SH-SY5Y cells of MPP^+^ + siTRIM3 group when compared with MPP^+^ + siNC group (*P* < 0.01) (Figure 4C).

TRIM3 effects on PI3K/AKT signal pathway in PD cell model was further explored. It could be noticed that, SH-SY5Y cells of MPP^+^ group had lower ratio of p-PI3K/PI3K and p-AKT/AKT than that of Control group (*P* < 0.01). Significantly higher ratio of p-PI3K/PI3K and p-AKT/AKT was found in SH-SY5Y cells of MPP^+^ + oeTRIM3 group when compared with MPP^+^ + oeNC group (*P* < 0.01). Instead, compared with MPP^+^ + siNC group, SH-SY5Y cells of MPP^+^ + siTRIM3 group showed remarkably lower ratio of p-PI3K/PI3K and p-AKT/AKT (*P* < 0.01 or *P* < 0.05) (Figure 4D).

### PI3K inhibitor treatment reversed the inhibitory effect of TRIM3 overexpression on PD cells apoptosis

**Figure 5:**
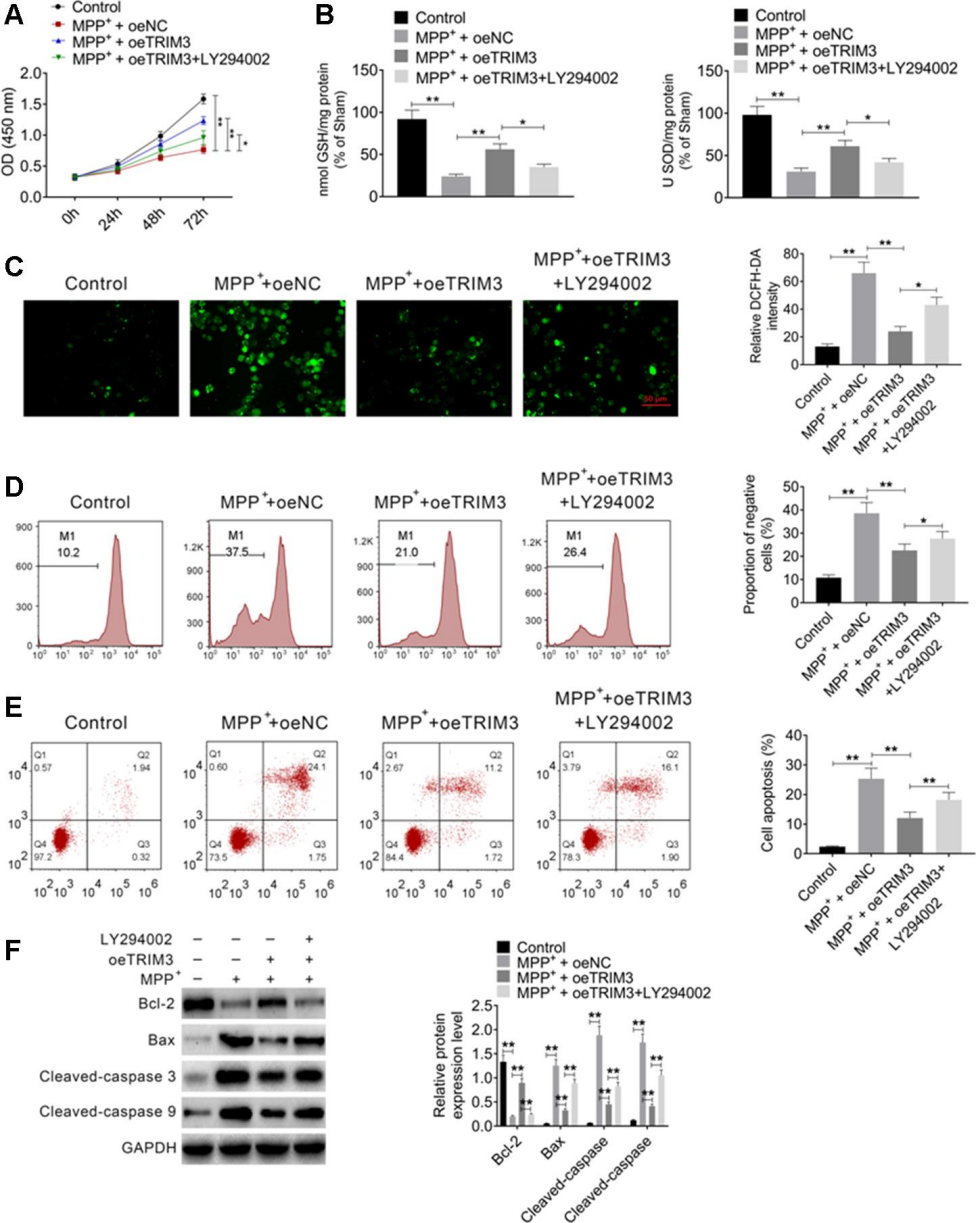
**PI3K inhibitor treatment reversed the inhibitory effect of TRIM3 overexpression on PD cells apoptosis.** (**A**) PI3K inhibitor treatment reversed the promoting effect of TRIM3 overexpression on PD cells proliferation. (**B**) PI3K inhibitor treatment reversed the promoting effect of TRIM3 overexpression on GSH and SOD secretion in PD cells. (**C**) PI3K inhibitor treatment reversed the inhibitory effect of TRIM3 overexpression on ROS level in PD cells. (**D**) PI3K inhibitor treatment reversed the inhibitory effect of TRIM3 overexpression on the MMP of PD cells. (**E**) PI3K inhibitor treatment reversed the inhibitory effect of TRIM3 overexpression on PD cells apoptosis. (**F**) PI3K inhibitor treatment reversed the effected of TRIM3 overexpression on apoptosis-related proteins expression in PD cells. * *P* < 0.05 and ** *P* < 0.01.

LY294002, a PI3K inhibitor, was used to treat SH-SY5Y cells. CCK-8 assay showed that, SH-SY5Y cells of MPP^+^ + oeNC group had significantly lower OD value than that of Control group (*P* < 0.01). When compared with MPP^+^ + oeNC group, much higher OD value was found in SH-SY5Y cells of MPP^+^ + oeTRIM3 group (*P* < 0.01). However, relative to MPP^+^ + oeTRIM3 group, the OD value of SH-SY5Y cells in MPP^+^ + oeTRIM3 + LY294002 group was obviously declined (*P* < 0.05) (Figure 5A). In terms of GSH and SOD level, SH-SY5Y cells of Control group exhibited markedly lower GSH and SOD level than that of MPP^+^ + oeNC group (*P* < 0.01). Compared with MPP^+^ + oeNC group, the GSH and SOD level of SH-SY5Y cells in MPP^+^ + oeTRIM3 group was obviously increased (*P* < 0.01). Conversely, in comparison with MPP^+^ + oeTRIM3 group, much lower GSH and SOD level was found in SH-SY5Y cells of MPP^+^ + oeTRIM3 + LY294002 group (*P* < 0.05) (Figure 5B).

The intracellular ROS was researched. As a result, remarkably higher relative DCFH-DA intensity was observed in SH-SY5Y cells of MPP^+^ + oeNC group when relative to Control group (*P* < 0.01). The relative DCFH-DA intensity was distinctly reduced in SH-SY5Y cells of MPP^+^ + oeTRIM3 group compared with MPP^+^ + oeNC group (*P* < 0.01). In comparison with MPP^+^ + oeTRIM3 group, SH-SY5Y cells of MPP^+^ + oeTRIM3 + LY294002 group showed prominently higher relative DCFH-DA intensity (*P* < 0.05) (Figure 5C). Afterword, the MMP of SH-SY5Y cells was monitored. The results showed dramatically higher negative cells proportion of MPP^+^ + oeNC group relative to Control group (*P* < 0.01). Simultaneously, remarkably lower negative cells proportion was found in SH-SY5Y cells of MPP^+^ + oeTRIM3 group when compared with MPP^+^ + oeNC group (*P* < 0.01). However, significantly elevated negative cells proportion was presented in SH-SY5Y cells of MPP^+^ + oeTRIM3 + LY294002 group in comparison with MPP^+^ + oeTRIM3 group (*P* < 0.05) (Figure 5D).

According to flow cytometry, relative to Control group, the apoptosis percentage was significantly increased in SH-SY5Y cells of MPP^+^ + oeNC group (*P* < 0.01). On the opposite, markedly decreased apoptosis percentage was shown in SH-SY5Y cells of MPP^+^ + oeTRIM3 group when compared with MPP^+^ + oeNC group (*P* < 0.01). However, SH-SY5Y cells of MPP^+^ + oeTRIM3 + LY294002 group had much higher apoptosis percentage than that of MPP^+^ + oeTRIM3 group (*P* < 0.05) (Figure 5E). Apoptosis-related proteins were monitored by Western blot. Notably, lower Bcl-2 protein expression and higher Bax, Cleaved-caspase 3 and Cleaved-caspase 9 proteins expression was occurred in SH-SY5Y cells of MPP^+^ + oeNC group when compared with Control group (*P* < 0.01). Oppositely, much increased Bcl-2 protein expression and decreased Bax, Cleaved-caspase 3 and Cleaved-caspase 9 proteins expression was observed in SH-SY5Y cells of MPP^+^ + oeTRIM3 group relative to MPP^+^ + oeNC group (*P* < 0.01). Interestingly, in comparison with MPP^+^ + oeTRIM3 group, distinctly diminished Bcl-2 protein expression and elevated Bax, Cleaved-caspase 3 and Cleaved-caspase 9 proteins expression was found in SH-SY5Y cells of MPP^+^ + oeTRIM3 + LY294002 group (*P* < 0.01) (Figure 5F).

## DISCUSSION

In this research, we detected the expression of TRIM3 in venous blood samples of PD patients. TRIM3 level in venous blood of PD patients was significantly decreased when compared to normal patients. The expression and function of TRIM3 in PD was further explored through successful establishment of PD mouse model and cell model. Results indicated that TRIM3 expression was severally reduced in PD mouse model and cell model. Up-regulation of TRIM3 could improve bradykinesia symptom of PD mice. In terms of mechanism, TRIM3 attenuated apoptosis in PD via activating PI3K/AKT signal pathway.

TRIM3 is located on cytoplasmic filaments and it is one of the members of the triple motif family. The functions associated with TRIM3, such as ubiquitin ligase activity, were found to be related to the nitrosative stress (an key factor for sporadic PD) [14]. This paper indicated that overexpression of TRIM3 could reduce apoptosis, MMP and the production of ROS in PD. PD is a common chronic progressive neurological degenerative disease, with a pathological feature characterized by the absence of dopaminergic neurons in the dense substantia nigra of the brain [15, 16]. By unremitting efforts of researchers, several major pathogenesis of PD have been revealed, including mitochondrial dysfunction, oxidative stress, neuroinflammatory response, excitotoxicity, apoptosis, etc. [17]. Oxidative stress response has been shown to promote neurotoxicity of dopaminergic neurons [18]. Most ROS is produced in mitochondria and mitochondrial dysfunction is closely related to the increased ROS production [19, 20]. Environmental factors, genetic factors and gene mutations in proteins associated with PD can result in mitochondrial dysfunction, which in turn promotes oxidative stress. The excessive ROS in the brain can lead to reactions such as nucleic acid disintegration and lipid peroxidation, thereby causing cell dysfunction and triggering dopaminergic apoptosis [20]. In addition, the excessive ROS also can result in the dysfunction of mitochondrial respiratory chain adenosine triphosphate synthesis and the release of ROS into the cytoplasm [21]. Excessive production of ROS inhibits the mitochondrial respiratory chain complex I. Unfortunately, deficiency of mitochondrial respiratory chain complex I is the main cause of apoptosis in the nervous system in patients with PD [22–24]. Research has found that the activity of mitochondrial respiratory chain complex I was reduced in the substantia nigra region of the brain in patients with sporadic PD [25]. ROS can also cause proteins to fold incorrectly, which promotes the neurodegenerative process of PD [26]. Thus, mitochondrial dysfunction and oxidative stress are considered to be the most prominent pathophysiological features of PD [27]. This article demonstrated for the first time that TRIM3 could inhibit apoptosis in PD. The mechanism might be through the inhibition of MMP and production of ROS in brain tissues.

Decreased GSH and SOD level was one of the pathological features for PD [28]. GSH and SOD are two important members of the free radical scavenging system. The anti-oxidant properties of GSH and SOD play a vital role in maintaining the oxidation and antioxidant balance in the body [29–31]. In this study, TRIM3 was found to elevate the level of GSH and SOD in PD mice midbrain tissues and PD cells. Furthermore, in PD cell model, up-regulation of TRIM3 increased Bcl-2 protein expression and decreased the expression of Bax, Cleaved-caspase 3 and Cleaved-caspase 9 proteins. Bcl-2 is well known for its anti-apoptotic function, whereas Bax has the function to promote apoptosis. Hong et al. [32] revealed that the down-regulation of Bcl-2 and up-regulation of Bax might participate in the early stage of PD. Mahsa et al. [33] pointed out that Bcl-2 might be involved in the pathogenesis of neuro degenerative diseases such as apoptosis. Previous data have demonstrated that the activated caspase-9 could cleave the caspase-3 precursor to produce the activated caspase-3. However, the onset of this apoptotic cascade reaction was one of the important causes of apoptosis [34]. Data from this paper indicated that TRIM3 could inhibit apoptosis in PD by promoting the expression of anti-apoptotic genes and suppressing the expression of pro-apoptotic related genes. Simultaneously, this study researched that TRIM3 increased the number of dopaminergic neurons (positive to TH) in the mice midbrain. As we all know, PD is accompanied by the loss of dopaminergic neurons, which is associated with resting tremors, rigidity, bradykinesia and postural instability [35]. TH is expressed by dopaminergic neurons and loss of TH-positive dopaminergic neurons is one of the main characteristics of PD [36, 37]. Data from this paper indicated that TRIM3 could inhibit the decrease of dopaminergic neurons number in the midbrain of PD rats.

At last but not the least, our data demonstrated for the first time that TRIM3 might attenuate apoptosis in PD through activating the PI3K/AKT signal pathway. In PD cell model, the overexpression of TRIM3 prominently reduced PD cells apoptosis and increased the phosphorylation of PI3K and AKT. However, LY294002 treatment (a PI3K inhibitor) reversed the inhibitory effect of TRIM3 overexpression on PD cells apoptosis. Nazca et al. [38] have illustrated the obviously over-activated anti-apoptotic PI3K/AKT signal pathway in PD, which might be to compensate for the increased oxidative stress and neuronal apoptosis. Some drugs have also been found to exert anti-apoptotic effects and protect dopaminergic neurons from neurotoxicity by activating thePI3K/AKT signal pathway [39, 40]. However, whether TRIM3 regulates PD development through regulating the PI3K/AKT signaling pathway has never been elucidated. This paper initially demonstrated that TRIM3 might attenuate apoptosis in PD through the activation of the PI3K/AKT signal pathway.

There was limitation in this research. The anatomical location of Tunel cells should be shown with low power magnification. However, due to the limitations of our laboratory, we are currently unable to conduct this experiment. In our future research, we will focus on studying whether Tunel cells are located in the ventral tegmental area (VTA) or in the substantia nigra reticulum (SNr).

Collectively, this research successfully constructed PD mouse model and PD cell model to research the function of TRIM3 in PD development. Results indicated that TRIM3 could suppress the MMP and production of ROS in PD. More importantly, TRIM3 could attenuate apoptosis in PD through the activation of the PI3K/AKT signal pathway. Thus, TRIM3 might be used as one of the potentially target for the treatment of PD. In the future, we will devote more researches to fully prove the feasibility of TRIM3 in the clinical treatment of PD.

## MATERIALS AND METHODS

### Blood samples

Venous blood samples were collected from 53 patients with PD and 53 healthy subjects. The average age of the 53 patients with PD was (65.8 w 9.7) years with the disease duration of (4.2 ± 1.6) years. Among the PD patients, 31 cases were male and 22 cases were female. The Hoehn-Yahr (H-Y) stage of patients was as follows: stage I (n = 6), stage II (n = 10), stage III (n = 17), stage IV (n = 11) and stage V (n = 9). For the 53 healthy participants, 29 participants were male and 24 participants were female with an average age of (62.3 ± 13.6) years. No statistically significant difference was found in age and gender between PD patients and healthy subjects. All participants have signed written informed consent in line with the approval by The Affiliated Brain Hospital of Nanjing Medical University ethics committee. The expression of TRIM3 mRNA in each venous blood sample was detected by quantitative real time polymerase chain reaction (qRT-PCR).

### Establishment of PD mouse model

All animal experiments involved in this research were conducted under the approval of animal ethics committee of The Affiliated Brain Hospital of Nanjing Medical University.

C57BL/6 mice (male, 10 weeks old, 20-25 g, n = 30) were provided from the Institute of Laboratory Animal Science, Chinese Academy of Medical Sciences (Beijing, China). Mice were kept in individual cages in a 12h light/dark cycle constant temperature room (25° C) with free access to food and water.

MPTP-HCl (Sigma-Aldrich, St. Louis, MO, USA) was used to construct PD mouse model. Briefly, mice (n = 24) were injected intraperitoneally with MPTP-HCl with a dose of 30 mg/kg/day for 4 consecutive days. The other 6 mice were served as Sham group and they were injected intraperitoneally with 0.9% sterile saline with an equivalent volume for 4 consecutive days. On the 1^st^, 3^rd^, 5^th^ and 7^th^ day after the last injection of MPTP-HCl, 6 mice were randomly selected and sacrificed to collect the midbrain. The midbrain was immediately stored at -80° C.

### Construction of TRIM3 overexpressed PD mouse model

C57BL/6 mice (male, 10 weeks old, 20-25 g, n = 24), also provided from the Institute of Laboratory Animal Science, Chinese Academy of Medical Sciences (Beijing, China), were randomly divided into 4 groups: Sham group, MPTP group, MPTP + oeNC group and MPTP + oeTRIM3 group. Six mice were contained in each group.

TRIM3 overexpression and negative control recombinant lentivirus vectors were established by GeneChem Co. Ltd (Shanghai, China). Mice of MPTP + oeNC group and MPTP + oeTRIM3 group were subjected to deep anesthesia with isoflurane in oxygen and nitrous oxide. Mice were then placed in a stereotactic frame. After the skull surface being exposed, a hole in the exposed skull was made to achieve needle positioning. Using a sterile syringe, mice were bilaterally injected with TRIM3 overexpression and negative control recombinant lentivirus vectors into the dorsal hippocampus (injection rate of 0.2 μL/min and injection duration of 10 min). Two days after injection, mice of the two groups were subjected to construction of PD mouse model via intraperitoneal injection with MPTP-HCl for 4 consecutive days (30 mg/kg/day) [3]. For mice of MPTP group, they were only injected intraperitoneally with MPTP-HCl with a dose of 30 mg/kg/day for 4 consecutive days. Mice of Sham group were only injected intraperitoneally with 0.9% sterile saline with an equivalent volume for 4 consecutive days. On the 5^th^ after the last injection of MPTP-HCl, all mice of the four groups were sacrificed. The midbrain was obtained and stored at -80° C [3].

### Pole test

The measurement of bradykinesia in mice was detected by pole test. Briefly, on the 1, 2, 3, 4 and 5 days after the last injection of MPTP-HCl, mice of each group were placed on a thick rod with a diameter of 8 mm and a height of 55 cm. The time of mice needed to turn around completely, climb down and reach the floor with four paws was recorded. The cut-off time for each test was 30 s.

### Tunel assay

The midbrain tissues from each mouse were fixed with 4% paraformaldehyde. After dehydration, the midbrain tissues were embedded with paraffin to prepare sections with a thickness of 4 μm. Protease K working solution (Solarbio, Beijing, China) was used to incubate these sections for 30 min at room temperature. H_2_O_2_ (3%) was then used to incubate sections for 10 min at room temperature to block endogenous peroxidase. Each section was incubated with 100 μL terminal transferase reaction solution (Solarbio, Beijing, China) at 37° C for 1 h in darkness. Subsequently, 50 μL horseradish peroxidase working solution (Solarbio, Beijing, China) was added onto sections for 30 min incubation at 37° C. Diaminobenzidine was used for color development and hematoxylin was used to counterstain the nucleus. After dehydration by gradient ethanol, the sections were treated with xylene and sealed in neutral resin. Tunel positive cells (green fluorescence) were observed under a fluorescence microscope. Five non-overlapping fields were randomly selected from each section to count the number of Tunel positive cells.

### Immunofluorescence

The number of dopaminergic neurons was measured by detecting tyrosine hydroxylase (TH) expression in midbrain of mouse. In brief, the midbrain of each mouse was cut into sections to a thickness of 5 μm. The sections were washed using Tris-buffered saline (TBS) and then incubated with TBS+ (0.3% Triton-X100 and 2% bovine serum albumin in TBS) for 45 min. Thereafter, primary antibody against TH (1:500, Boster, Wuhan, China) was used to incubate the sections for 48 h. After being washed by TBS, the sections were incubated with FITC-conjugated secondary antibody (1:500, Boster, Wuhan, China) for 3 h. The TH immunopositive dopaminergic neurons were observed under fluorescence microscope with red fluorescence as positive TH expression signals [41].

### Cell culture and construction of PD cell model

Human neuroblastoma cell line SH-SY5Y was purchased from the American Type Culture Collection (Manassas, VA, USA). Cells were maintained in Dulbecco's modified Eagle medium (DMEM) containing 10% fetal bovine serum (FBS) in a constant temperature incubator at 37° C, 5% CO_2_. At the logarithmic growth phase, cells were collected to establish PD cell model by treated with MPP^+^ solution (0.25, 0.5 and 1 mM) (Sigma-Aldrich, St. Louis, MO, USA) for 24 h. SH-SY5Y cells only cultured in DMEM containing 10% FBS were used as Control group.

### Cell transfection and treatment by MPP^+^ solution and LY294002

SH-SY5Y cells were dispersed in serum-free DMEM with a density of 1 * 10^5^ cells/mL. Then cells were seeded in 6-well plates with 1 mL of cell suspension in each well. TRIM3 overexpression vector and negative expression vector, as well as TRIM3 siRNA (sequence: 3'-TATGCAGTATCTGCCTGGATCGGTA-5') and negative control (3'-TATTGACTACGTGTCTAGGCGCGTA-5') (all provided by GenePharma, Shanghai, China) were respectively used to transfect SH-SY5Y cells using Lipofectamine 2000 Reagent (Thermo Fisher Scientific, USA). Cells are sequentially named oeTRIM3 group, oeNC group, siTRIM3 group and siNC group. All the transfected SH-SY5Y cells were transferred to DMEM with 10% FBS after 8 h of transfection. After 24 h incubation in DMEM with 10% FBS at 37° C, 5% CO_2_, the transfection efficiency was detected by qRT-PCR.

To establish PD cell model, cells of the above four groups were then treated with DMEM containing 10% FBS and 1 mM MPP^+^ solution for 24 h. After treated by MPP^+^ solution, these cells were further set as MPP^+^ + oeTRIM3 group, MPP^+^ + oeNC group, MPP^+^ + siTRIM3 group and MPP^+^ + siNC group respectively. In addition, SH-SY5Y cells treated with DMEM containing 10% FBS and 1 mM MPP^+^ solution were served as MPP^+^ group. SH-SY5Y cells only cultured in DMEM containing 10% FBS were used as Control group.

In addition, LY294002, a PI3K inhibitor, was used to treat SH-SY5Y cells. Briefly, SH-SY5Y cells, which were transfected by TRIM3 overexpression vector, were collected and treated by DMEM (with 10% FBS) containing 1 mM MPP^+^ solution and 10 μM LY294002 (PI3K inhibitor) for 24 h. These cells were set as MPP^+^ + oeTRIM3 + LY294002 group.

### Detection of glutathione (GSH) and superoxide dismutase (SOD) level

Mice midbrain tissues were stirred to prepare homogenate. Each homogenate sample was centrifuged at 3000 r/min for 15 min. Supernatant from each sample was collected. Equal amount of each supernatant sample was subjected to the detection of GSH and SOD level by using Kit (Beyotime, Shanghai, China) according to the instructions. In addition, SH-SY5Y cells of each group were lysed and centrifuged to obtain the supernatant. Equal amount of each supernatant sample was used to detect the GSH and SOD level as described above.

### Detection of intracellular reactive oxygen species (ROS) and mitochondrial membrane potential (MMP)

For the detection of intracellular ROS, a total of 1 * 10^6^ SH-SY5Y cells of each group were collected and washed three times with PBS. Dichlorodihydrofluorescein diacetate (DCFH-DA) (10 μL) was added to incubate cells for 30 min at 37° C. After washed with PBS, DCFH-DA intensity of each group were observed and analyzed using a laser confocal scanning microscope.

The MMP of SH-SY5Y cells in each group were also measured. Briefly, 1 * 10^6^ SH-SY5Y cells of each group were harvested and washed with PBS. Then these cells were incubated with rhodamine solution (10 μL) for 30 min at 37° C in darkness. PBS was used to wash cells for three times. The MMP of cells was measured by flow cytometry and the proportion of negative cells was analyzed.

### Cell counting kit-8 (CCK-8) assay

SH-SY5Y cells were seeded into 96-well plates and maintained in DMEM containing 10% FBS and MPP^+^ (0, 0.25, 0.5 and 1 mM). Cells were cultured at 37° C, 5% CO_2_ for 48 h. CCK-8 reagent (10 μL) was then added into each well for 2 h incubation at 37° C. The optical density (OD) value of each well was measured using a microplate reader at a wavelength of 450 nm.

Furthermore, SH-SY5Y cells of each group were also incubated for 48 h in 96 well plates in their respective culture medium. The OD value of each well was detected at a wavelength of 450 nm as described above.

### Flow cytometry analysis

SH-SY5Y cells of each group were harvested and dispersed in the annexin V binding buffer. According to the manual of the Annexin V-FITC Apoptosis Detection Kit (Beyotime, Shanghai, China), cells of each group were subjected to 15 min staining with Annexin V-FITC (5 μL) and propidium iodide (5 μL) at room temperature. The apoptosis of cells in each group was analyzed using flow cytometry.

### qRT-PCR

Total RNA in venous blood samples, midbrain tissues of mice and SH-SY5Y cells was extracted by using TRizol reagent (Life Technologies, MD, USA). cDNA synthesis kit (Beyotime, Shanghai, China) was used to perform reverse transcription reaction to synthesize cDNA templates. qRT-PCR was carried out using the Power SYBR GREEN PCR master mix (Applied Biosystems, CA, USA) with the 7000 Sequence Detection System (Applied Biosystems, CA, USA). The reaction parameters were as follows: 95° C for 10 min, and 40 cycles at 95° C for 15 s and 60° C for 30 s. The primer sequences were as follows: TRIM3, forward: 5'-GGCTGACTGGGGCAACAGCCGCATC-3', reverse: 5'-ATCTGCAGAACCACTGTATGGTCCA-3'. GAPDH, forward: 5'-GAAGGTGAAGGTCGGAGTC-3', reverse: 5'-GAAGATGGTGATGGGATTTC-3'. The relative expression of TRIM3 mRNA was normalized to GAPDH and calculated by 2-ΔΔCt method.

### Western blot

Total proteins in mice midbrain tissues and SH-SY5Y cells were collected using lysis buffer (Beyotime, Shanghai, China). Each protein sample was quantified by using the BCA Kit (Beyotime, Shanghai, China). A total of 50 μg of each protein sample was subjected to 10% sodium dodecyl sulphate polyacrylamide gel electrophoresis (SDS-PAGE). Then proteins were transferred to polyvinylidene difluoride (PVDF) membrane. After incubated with 5% skimmed milk, the PVDF membrane was sequentially incubated with primary antibodies for 12 h at 4° C and horseradish peroxidase (HRP)-conjugated secondary antibody for 1 h at 37° C. The primary antibodies used in this research were as follows: primary antibodies against TRIM3, Bcl-2, Bax, Cleaved-caspase 3, Cleaved-caspase 9 proteins, p-PI3K, PI3K, p-AKT and AKT (1:1000, Abcam, Cambridge, MA, USA). The secondary antibody (Cell Signaling Technology, Danvers, MA, USA) was diluted to a ratio of 1: 2000. GAPDH was set as the internal control. Finally, enhanced chemiluminescence (Pierce, Rockford, IL) was used to visualize the blots.

### Statistical analysis

In this study, quantitative data was presented in the form of mead ± standard deviation (SD). Data was processed using SPSS 19.0 software (Chicago, IL, USA). Student's t-test was used to analyze the difference between two independent groups. Analysis of Variance (ANOVA) was selected to analyze the difference at least in three groups. *P* < 0.05 meant a statistically significant difference.

### Ethics approval

Patient samples were collected with written informed consent in accordance with the Declaration of Helsinki and in a process approved by the Ethical Committee of The Affiliated Brain Hospital of Nanjing Medical University (approval number: EC201812014).
